# Impact of China's national drug price negotiation on pharmaceutical firms' innovation activities

**DOI:** 10.3389/fpubh.2026.1812550

**Published:** 2026-05-11

**Authors:** Heng Xu, Jingyu Wang, Qian Zhou

**Affiliations:** School of International Pharmaceutical Business, China Pharmaceutical University, Nanjing, China

**Keywords:** Innovation investment, innovation performance, Multi-period difference-in-differences model, National drug price negotiation, pharmaceutical firms

## Abstract

**Background:**

The National Drug Price Negotiation (NDPN) is a key policy in China for controlling healthcare costs and improving drug accessibility. However, its impact on pharmaceutical firms' innovation remains controversial, with limited empirical evidence on micro-level mechanisms.

**Methods:**

Using panel data of Chinese A-share listed pharmaceutical companies from 2010 to 2024, we constructed a multi-period difference-in-differences (DID) model to empirically analyze the causal effects, transmission mechanisms, and heterogeneity of negotiation access on pharmaceutical firms' innovation activities. Identification robustness was enhanced through a series of robustness tests including parallel trend tests, placebo tests, and a combination of Propensity Score Matching and Differences-in-Differences (PSM-DID).

**Results:**

Negotiation access significantly stimulated firms' innovation investment. Mechanism tests indicated the effect was driven by the external signal transmission channel, through enhanced capital market valuation and government subsidies, while the internal resource release channel was not effectively realized, as reductions in marketing intensity did not significantly increase innovation investment. Heterogeneity analysis showed stronger effects in large firms and those with greater market power, but with attenuated effects for firms negotiating shortly after drug launch. Further analysis revealed a structural divergence between strategic and substantive innovation: non-invention patent quantity increased without quality improvement, whereas invention patent quality improved despite insignificant changes in quantity.

**Conclusions:**

NDPN positively promotes innovation but with structural heterogeneity. Policies should maintain a value-oriented negotiation mechanism, ensure smooth external signal transmission, facilitate internal resource reallocation, support weaker firms and early-stage products, improve clinical access to achieve dual goals of “ensuring coverage” and “promoting innovation.”

## 1 Introduction

Pharmaceutical innovation activity serves as the core driver for advancing high-quality development in the pharmaceutical industry and achieving high-level scientific and technological self-reliance. As a strategic industry vital to national economy, people's livelihood, and public health, biomedical innovation not only directly determines the market competitiveness of pharmaceutical companies but also provides critical support for safeguarding public health and advancing the “Healthy China” initiative. Under China's current healthcare system, pharmaceuticals possess quasi-public goods attributes, with the basic medical insurance fund serving as their largest purchaser. Consequently, decisions regarding drug inclusion in the reimbursement list and payment standards profoundly affect pharmaceutical firms' market returns and profit models, fundamentally shaping their intrinsic motivations and long-term incentives for innovation activities (1).

To balance the need for effective control over the rapid growth of healthcare costs with the sustained encouragement of innovation in the pharmaceutical industry, China has explored and established NDPN as a strategic purchasing mechanism since 2016. Initially led by the former National Health and Family Planning Commission, the negotiations were transferred to the Ministry of Human Resources and Social Security in 2017. After the establishment of the National Healthcare Security Administration in 2018, drug price negotiations entered a regularized and standardized stage, with a dynamic adjustment mechanism implemented on an annual basis. By December 2025, China has completed 10 rounds of national drug price negotiations, through which 719 pharmaceutical products (by generic name) were successfully added to the National Reimbursement Drug List (NRDL) (Table 1). Researches indicate that the drug price negotiation policy has significantly reduced drug prices, substantially alleviating patients' medication burdens, and effectively controlling per capita medical expenses and medical insurance fund expenditures (2). It has markedly improved drug accessibility and utilization rates in numerous areas, including anticancer drugs (3, 4) and chronic disease medications (5), thereby enhancing the equity and accessibility of healthcare services. This has positively impacted patient health outcomes, fully validating the public welfare policy objective of “ensuring basic coverage” (6). However, whether this mechanism can simultaneously achieve the policy goal of “promoting innovation”—that is, whether firms are incentivized to sustain innovation investment despite sharp price reductions for negotiated drugs—remains debated in practice. Innovation investment refers to the direct expenditures on pharmaceutical innovation activities, such as new drug development and clinical trials, and is a key item disclosed in the annual reports of Chinese listed firms. In 2019, Jilin Aodong Pharmaceutical Group's exclusive product, DuzhiWan, was included in the reimbursement list through negotiation, yet its 2020 annual report showed a 40.41% decline in research and development (R&D) expenditure compared with 2019. Similarly, in 2023, LIVZON's new product, Triptorelin Acetate Microspheres for Injection, was included in the reimbursement list through negotiation. However, the company's operating revenue decreased by 4.97% in the following year. The R&D expenditure fell from 1.235 billion CNY in 2023 to 1.044 billion CNY, a decrease of approximately 15.45%. In addition, in 2022, at least 13 innovative drugs eligible for negotiation temporarily chose not to participate in the drug price negotiation. These real-world cases illustrate the strategic choices Chinese pharmaceutical companies face regarding drug price negotiations, providing concrete evidence of the potential “inhibition effect” on innovation that negotiations may generate.

**Table 1:** National drug price negotiation overview.

Negotiation batch	Responsible department	Negotiation Year	Release time of the national reimbursement drug list (NRDL)	Number of newly added drugs to the national reimbursement drug list
Batch 1	National Health and Family Planning Commission of the People's Republic of China	2016	February 2017	3
Batch 2	Ministry of Human Resources and Social Security of the People's Republic of China	2017	July 2017	36
Batch 3	National Healthcare Security Administration	2018	October 2018	17
Batch 4		2019	January 2020	70
Batch 5		2020	March 2021	96
Batch 6		2021	January 2022	67
Batch 7		2022	January 2023	108
Batch 8		2023	January 2024	121
Batch 9		2024	January 2025	89
Batch 10		2025	January 2026	112

**Data source:**

Notice of the ministry of human resources and social security on issuing the national basic medical insurance, work-related injury insurance and maternity insurance drug list (2017 Edition). (https://www.mohrss.gov.cn/xxgk2020/fdzdgknr/zlbmxgwj/ylbx/201702/t20170223_266775.html).

Notice of the ministry of human resources and social security on including 36 drugs into category b of the national basic medical insurance, work-related injury insurance and maternity insurance drug list. (https://www.mohrss.gov.cn/xxgk2020/fdzdgknr/zlbmxgwj/ylbx/201707/t20170718_274153.html).

Notice of the national healthcare security administration on including 17 Anti-cancer Drugs into Category B of the national basic medical insurance, work-related Injury Insurance and Maternity Insurance Drug List. (https://www.gov.cn/zhengce/zhengceku/2018-12/31/content_5438693.htm).

Joint notice of the national healthcare security administration and the ministry of human resources and social security on issuing the national basic medical insurance, work-related injury insurance and maternity insurance drug list (2019–2023). (https://www.nhsa.gov.cn/).

Current academic discussions on whether price negotiations effectively promote pharmaceutical firms' innovation remain limited and require further investigation. On the one hand, as a demand-side policy, NDPN operates primarily through a “price-for-volume” mechanism, expanding market space and enhancing expected returns, thereby stimulating firms' innovation incentives (7). For instance, Zhu et al. (1) found that price negotiations significantly increased firms' R&D investment and patent applications, while Liao et al. (8) showed that negotiations could incentivize innovation through demand-pull effects and talent attraction. On the other hand, mandatory price reductions driven by market mechanisms may distort R&D incentives. If short-term price cuts cannot be offset by volume growth, firms' profit margins are squeezed, potentially reducing innovation investment, delaying new therapy launches, and even causing structural deficiencies in innovation motivation (9).

Overall, existing studies have several limitations: first, mechanisms through which price negotiations affect innovation are not fully explored; second, the boundary conditions of policy effects, such as firm market power and negotiation timing, remain insufficiently examined; third the characterization of innovation performance is relatively narrow, with limited attention to the distinction between strategic and substantive innovation under the policy environment.

To address these gaps and enrich existing findings, we use the eight rounds of national drug price negotiations implemented in China from 2016 to 2023 as a quasi-natural experiment to empirically examine the effects and mechanisms of price negotiations on pharmaceutical firms' innovation activities. The potential contributions are fourfold. First, in terms of methodology, we employ a multi-period difference-in-differences model on a sample of Chinese listed pharmaceutical firms from 2010 to 2024, aiming to identify the net effect of price negotiations on innovation investment more precisely and over a longer horizon, thereby expanding micro-level empirical evidence in this field. Second, regarding theoretical mechanisms, we investigate the impact pathways through external signal transmission and internal resource release, deepening understanding of how “strategic purchasing” of national medical insurance fund incentivizes industrial innovation. Third, in terms of heterogeneity analysis, we reveal the conditional and structural variation of policy effects, showing differentiated impacts of price negotiations across firm size, market power, and negotiation timing, enriching research on the boundary conditions of policy effects. Fourth, we extend the analysis to innovation performance, examining how the policy affects strategic vs. substantive innovation. These findings provide detailed empirical guidance for optimizing negotiation rules and achieving long-term synergy between “ensuring coverage” and “promoting innovation.”

## 2 Theoretical analysis and research hypotheses

### 2.1 Institutional context and direct effect hypothesis

The pharmaceutical industry is characterized by high investment, high risk, long cycles, and significant public goods attributes. Innovation activities often face “market failure” due to positive externalities. According to neoclassical economic theory, government policy intervention is crucial to correct spillover effects from innovation and incentivize R&D (10). Currently, China's pharmaceutical industry is undergoing a strategic transformation from “primarily generic-based” to “innovation-driven.” During this process, the government directly influences corporate investment activities and strategic decisions through a series of policy tools that primarily fall into two categories: supply-side incentives and demand-side pull mechanisms (11). On the supply side, policies such as R&D subsidies (12) and intellectual property protection (13) have been extensively studied and proven effective in promoting pharmaceutical companies‘ innovation investment. Regarding demand, drug price negotiation and volume-based procurement (VBP) serve as the two core mechanisms through which the National Healthcare Security Administration exercises its strategic purchasing function, jointly playing a pivotal role in regulating market access for pharmaceuticals and guiding market price formation (14). However, existing research has predominantly focused on VBP policy, examining its impact on drug prices, sales volumes (15) as well as manufacturers' innovation (16). Studies on how drug price negotiations influence corporate innovation, along with its underlying mechanisms and heterogeneous effects, remain relatively scarce.

Unlike VBP, NDPN focuses on high-priced patented drugs, exclusive products, and innovative medicines. These are entry-based negotiations for inclusion in the reimbursement list, primarily targeting pharmaceutical firms engaged in innovative drug R&D that have drugs under development or already on the market. Essentially, the process constitutes a contract between the insurance authority and innovative drug firms, with “inclusion in the National Reimbursement Drug List” as the core consideration and “expected national market share” as the performance target. Accompanying payment standards and hospital evaluation policies provide a clear entry channel and reimbursement guarantee, aiming to significantly enhance market accessibility and potential sales through a “price-for-volume” mechanism (17). However, successful negotiations do not imply immediate availability in hospital pharmacies. Multiple barriers remain between “list inclusion” and “clinical accessibility,” including hospital pharmacy committee review cycles, pre-allocation constraints on insurance budgets, physicians' prescribing habits, and drug distribution logistics, forming the “last-mile” challenge for negotiated drugs (1). If volume growth lags or falls short of expectations, firms face the dual pressure of “price reduced, volume not realized,” potentially weakening or delaying the innovation incentive effect.

Nevertheless, as a milestone policy in China's healthcare reform, NDPN provides an institutional foundation to incentivize innovation investment by innovative drug firms. From a demand-pull perspective, negotiations reduce drug prices while granting reimbursement eligibility, significantly lowering patient payment barriers compared with the out-of-pocket market. This enhances expected market demand, expands national market opportunities for innovative products, and improves the prospective returns on innovation, directly strengthening firms' core innovation incentives. From a quality-selection perspective, under policy guidance prioritizing cost control and clinical value, the negotiation mechanism may create a structural screening effect, enabling drugs with high clinical value that address unmet medical needs to gain stronger bargaining power and sustainable market advantages (14). This encourages firms to focus R&D on high-investment, high-risk substantive innovation, leveraging product uniqueness to secure quality premiums and innovation returns, thereby forming a virtuous cycle of “innovation investment—clinical recognition—market returns—further innovation investment”.

Based on the above analysis, we propose the following hypothesis:

**H1: Inclusion of a firm's drugs in the National Reimbursement Drug List through price negotiations will positively incentivize pharmaceutical firms' innovation investment**.

### 2.2 Mechanism hypothesis from the external signal transmission perspective

From the perspective of external signal transmission, signaling theory posits that in markets characterized by information asymmetry, high-quality firms must undertake specific, observable, and costly actions to convey credible signals about their quality or future prospects to external parties at an informational disadvantage, such as investors or government agencies (18). Successful inclusion in the NRDL not only facilitates hospital market access for the product but may also serve as a strong “certification signal” to capital markets and government authorities. This signal conveys multiple favorable pieces of information: first, the drug's clinical value and pharmacoeconomic attributes have passed rigorous evaluation by the expert panel of the National Healthcare Security Administration; second, the firm's commercialization capabilities and pricing strategy have undergone national-level scrutiny (8). Such publicly observable information helps reduce information asymmetry between the firm and external capital, mitigating uncertainty about the firm's future profitability.

On this basis, successful price negotiation may improve the external financing environment for corporate innovation investment through two channels:

First, enhancing capital market valuation. Authorization for reimbursement provides authoritative endorsement, reducing information asymmetry between investors and the firm, and triggering market reassessment of the firm's value. Higher valuation can enhance equity financing capacity, lower the cost of external capital, improve expectations of future cash flows, and strengthen management's confidence and commitment to long-term innovation strategies (19). Improved market expectations may create a favorable capital environment for sustained high-intensity innovation investment.

Second, increasing government subsidies. When allocating funding, government agencies often face difficulty directly assessing firms' true innovation quality and project feasibility (20). Successful price negotiation serves as a precondition or merit factor in technology project applications and may act as a national-level quality certification, signaling that the firm possesses R&D capabilities, regulatory compliance, recognized clinical value, and commercialization capacity. This reduces government screening costs and increases the likelihood that negotiated firms receive priority consideration in competitive project evaluations, thereby enhancing access to government subsidies and policy financing. Government support not only alleviates R&D funding constraints but may also further transmit positive signals to capital markets, creating a dual signaling synergy (21).

Based on the above theoretical analysis, we propose the following hypothesis:

**H2: Inclusion of a firm's drug in the National Reimbursement Drug List through price negotiations can incentivize firms' innovation investment by enhancing capital market valuation and increasing government subsidies**.

### 2.3 Mechanism hypothesis from the internal resource release perspective

From the perspective of internal resource release, the resource-based view (RBV) posits that a firm's competitive advantage stems from heterogeneous resource endowments and the efficiency of resource allocation, with core competencies built through effective integration and utilization of internal resources (22). Inclusion in the reimbursement list alters firms' revenue generation logic—from “high marketing-driven” to “insurance reimbursement-driven”—restructuring the opportunity cost of resource allocation. Once negotiated drugs gain reimbursement eligibility, firms can theoretically rely on the institutional guarantee of insurance payments to expand market demand, reducing dependence on traditional marketing efforts and thereby releasing financial resources (23).

According to the RBV concept of resource flexibility, firms have the ability to reallocate resources, and the ultimate deployment depends on managerial decisions and external constraints (24). Freed marketing resources can be used for debt repayment, shareholder returns, capacity expansion, cash reserves, or, under innovation-oriented strategic guidance, invested in R&D to support internal funding for innovation.

Based on this theoretical analysis, the following hypothesis is proposed:

**H3: Inclusion of a firm's drug in the National Reimbursement Drug List through price negotiations can incentivize firms' innovation investment by reducing marketing intensity**.

## 3 Study design

### 3.1 Data sources

The release of the “Opinions on Deepening the Reform of the Medical and Health System” on March 17, 2009, marked the launch of a new round of healthcare reform in China. We define the observation window from 2010 (the first full year after the implementation of the healthcare reform) to 2024, covering the period from the first NDPN in 2016 to the eighth round in 2023. The period from 2010 to 2015 was defined as the policy baseline for the following reasons: first, it meets the identification requirements for DID model by providing a six-year pre-policy window for the parallel trends assumption. Second, it ensures a relatively “clean” baseline, as VBP had not yet been implemented and other major policy interventions were minimal. Third, it aligns with the long R&D cycle in the pharmaceutical industry, facilitating an accurate depiction of firms' innovation activities.

We select listed pharmaceutical companies on the Shanghai and Shenzhen A-share markets as the research sample, based on several considerations. First, the A-share market aggregates mainstream Chinese pharmaceutical firms and represents the primary targets of the NDPN. Second, listed companies disclose information according to standardized regulations, ensuring high data availability on operations and innovation. Third, the sample encompasses firms of varying sizes and innovation capacities, including both generic and innovative drug manufacturers, thereby supporting diversified empirical analyses. The sample scope is defined according to the “C27” pharmaceutical manufacturing category in the National Economic Industry Classification (GB/T 4754) and supplemented through manual verification based on the industry classification standard system released by Shenyin Wanguo Securities Co., Ltd. in July 2021.

Data processing proceeded as follows: (1) ST and ^*^ST firms were excluded; (2) observations with missing control variables were removed; (3) all continuous variables were winsorized at the 1% level at both tails to control for extreme values. The final sample included 333 A-share listed pharmaceutical companies, comprising 60 biopharmaceutical firms in the treatment group—whose products were successfully included in the NRDL via price negotiations between 2016 and 2023—and 273 firms in the control group whose products were not included during the same period.

The core variable, “whether a firm has drugs included in the National Reimbursement Drug List through price negotiations” was sourced from the ByDrug database, supplemented by manual verification using the PharmaOne database from the China Pharmaceutical Industry Information Center and Mininet database. All other firm-level data were sourced from the China Stock Market & Accounting Research Database (CSMAR).

### 3.2 Variable definition

Dependent Variable: The dependent variable in this study is corporate innovation investment (RD). Existing literature typically measures innovation investment using either R&D expenditure as a share of revenue or as a share of total assets (8, 25). Considering that the pharmaceutical industry is highly policy-intensive, where regulatory shifts may significantly impact operating revenue, leading to greater volatility in operating revenue (Supplementary Figure 1), revenue-based measures may be subject to short-term fluctuations and fail to accurately capture firms' innovation investment. Following Umar et al. (26), we measure innovation investment as the ratio of R&D expenditure to total assets, reflecting firms' allocation of R&D resources relative to their asset size.

Independent Variable: The explanatory variable in this study is “whether the firm has drugs included in the National Reimbursement Drug List through price negotiations” (nego_dum). Given that NRDL negotiation results typically take effect in the following year (e.g., the 2021 negotiation results implemented on January 1, 2022), to ensure alignment between the policy timing and firm innovation investment, we defined the year following a company's successful drug negotiation as the starting year for the policy shock. The explanatory variable (nego_dum) was set to 1 starting from this year and 0 for all preceding years. For companies with multiple drugs entering the list, following the approach of Liao et al. (8), the year of the first entry was defined as the policy shock, with the variable set to 1 for subsequent years to satisfy the persistence requirement of the DID model. Additionally, if a firm was included in the NRDL but was not publicly listed, the data were aggregated to the parent company level when the parent was listed, ensuring accurate identification of the treatment status.

Mediating Variables: To examine the mechanisms of external signal transmission and internal resource release, we selected the following three mediating variables:

External Capital Market Valuation (TobinQ): Tobin's Q is defined as the ratio of a company's market value to the replacement cost of its assets, capturing adjustments in external capital markets regarding the firm's value and growth expectations (27). It serves as a concrete indicator of investors' reassessment and recognition of long-term investment opportunities and growth potential (28). Tobin's Q was used to measure external capital market valuation.

Government subsidies (Gov): Government subsidies is an important indicator of the level of fiscal support a firm receives. Following Liao et al. (8), subsidies were measured as the natural logarithm of “the amount received plus one”, mitigating the impact of skewed data distributions.

Marketing intensity (SalesExp): Marketing intensity reflects the extent of a firm's innovation investment in market promotion and serves as a key indicator of internal resource allocation. We measured it as the ratio of selling expenses to operating revenue to capture how negotiations affect firms' operational strategies, which in turn influences their allocation of resources to innovation. Using a different denominator from that of the dependent variable (RD) also helps mitigate potential spurious correlations (29).

Control Variables: To accurately identify the net effect of the NDPN on innovation investment, Following Tan (23), we selected control variables across firm characteristics, financial structure, corporate governance, operational efficiency, and macroeconomic environment. Specifically, firm size (Size) and listing age (ListAge) reflect firms' resource endowments and life cycle stage, serving as fundamental factors influencing innovation investment; Leverage (Lev) controls for financial risk, as higher debt levels may suppress innovation through risk-taking mechanisms; Ownership concentration (Top1) and institutional ownership ratio (INST) capture the effect of corporate governance on R&D decisions from the perspective of equity structure and external monitoring; Asset turnover (ATO) measures operational efficiency, reflecting asset utilization, while the proportion of fixed assets (FIXED) controls for potential R&D crowding-out effects due to differences in asset structure; In addition, value added of the secondary industry (Indus) was included as a proxy for the macroeconomic environment, controlling for regional industrial structure and economic development levels that could influence pharmaceutical innovation. Variable definitions are presented in Table 2.

**Table 2:** Definitions of relevant variables.

Variable type	Variable name	Variable symbol	Measurement method
Dependent variable	Corporate innovation investment	RD	Corporate R&D expenditure/Total assets
Independent variable	Whether the enterprise has drugs included in the National through negotiation	nego_dum	Assigned a value of 1 for the implementation year of the catalog and subsequent years if the enterprise has drugs included in the National Reimbursement Drug List through price negotiations; otherwise 0
Mediating variable	Capital Market Value	TobinQ	Market value of the company's stock/Replacement cost of the company's assets
	Government Subsidy	Gov	ln (Government Subsidies+1)
	Marketing Investment Intensity	SalesExp	Selling Expenses/Operating Revenue
Control variable	Firm Size	Size	Natural logarithm of total assets
	Listing Age	ListAge	ln (Current year-Listing year+1)
	Asset-Liability Ratio	Lev	Total liabilities/Total assets
	Institutional Ownership Ratio	INST	Institutional investor shareholding ratio/100
	Largest Shareholder's Shareholding Ratio	Top1	The largest shareholder's shareholding ratio/100
	Asset Turnover Ratio	ATO	Net operating revenue/Average total assets
	Fixed Asset Ratio	FIXED	Net fixed assets/Total assets
	Value-Added of the Secondary Industry	Indus	ln(value-added of the secondary industry in 100 million yuan)

### 3.3 Model setting

The multi-period difference-in-differences (DID) model identifies the net effect of successful drug price negotiations on corporate innovation investment by comparing the differences in changes between the treatment and control groups before and after policy implementation (30, 31). Given the time lag in drug price negotiations for drugs from different companies, we constructed a DID model, as shown in Equation (1).

$$\begin{array}{l} RD_{it}=\alpha_{0}+\beta {nego\text{\_}dum}_{it}+\tau X_{it}+\lambda_{i}+\theta_{t}+\mu_{p}+\varepsilon_{it} \end{array}$$ (1)

where subscripts *i* and *t* denote firm and year, respectively. *RD*_*it*_ is the dependent variable, indicating firm's innovation investment in year t. *nego*_*dum*_*it*_ serves as the core independent variable, with its coefficient β reflecting the impact of a firm's drug being included in the NRDL through negotiations on innovation investment. *X*_*it*_ constitutes a set of control variables. λ_*i*_, θ_*t*_, μ_*p*_ denote firm fixed effect, year fixed effect, and province fixed effect respectively. ε_it_ represents the random error term. α_0_ is the intercept term. Standard errors were clustered at the firm level to account for potential serial correlation and heteroskedasticity.

## 4 Empirical analysis

### 4.1 Descriptive statistics

Table 3 presents descriptive statistics of the key variables. The mean value of the dependent variable (RD) is 3.08%, which is consistent with the pharmaceutical manufacturing sector's high innovation investment profile. Its large standard deviation and wide range (from 0.11% to 20.76%) indicate significant variation in innovation investment intensity across firms. The mean value of the core explanatory variable (nego_dum) is approximately 0.071, meaning that only approximately 7.1% of the sample enterprises gained access to the NRDL through negotiations during the sample period.

**Table 3:** Results of descriptive statistics.

Variable name	Observations	Mean	Standard deviation	Min	Max
RD	3012	0.0308	0.0306	0.0011	0.2076
nego_dum	3012	0.0710	0.2569	0.0000	1.0000
TobinQ	3012	2.4902	1.5952	0.9362	9.5840
Gov	3012	15.9696	3.0890	0.0000	19.2173
SalesExp	3012	0.2497	0.1736	0.0072	0.7384
Size	3012	21.9941	0.9861	19.8522	24.5022
ListAge	3012	2.0869	0.9297	0.0000	3.4012
Lev	3012	0.3050	0.1767	0.0358	0.8209
INST	3012	0.4423	0.2337	0.0097	0.9067
Top1	3012	0.3272	0.1350	0.0820	0.6922
ATO	3012	0.5404	0.2730	0.0622	1.4724
FIXED	3012	0.2080	0.1116	0.0159	0.5258
Indus	3012	9.4501	1.0076	6.3677	10.9883

Correlations among the variables were examined to eliminate potential interference from multicollinearity issues in the model estimation (Table 4). The Pearson correlation coefficients revealed that the correlation coefficients between variables were well below the empirical threshold of 0.5, indicating that no highly linear relationships existed among the variables. Multicollinearity diagnostics were conducted using the variance inflation factor (VIF). The results showed that all variables exhibited low VIF values, with an average of 1.32 and a maximum of 2.00, both below the commonly used threshold of 5. This indicates that there were no severe multicollinearity issues among the explanatory variables.

**Table 4:** Pearson correlation analysis.

Variable	RD	nego_dum	TobinQ	Gov	SalesExp	Size	ListAge	Lev	INST	Top1	ATO	FIXED	Indus
RD	1.000												
nego_dum	0.330^***^	1.000											
TobinQ	0.166^***^	0.019	1.000										
Gov	0.165^***^	0.146^***^	−0.074^***^	1.000									
SalesExp	0.294^***^	0.166^***^	0.050^***^	0.035^*^	1.000								
Size	0.006	0.213^***^	−0.240^***^	0.340^***^	−0.133^***^	1.000							
ListAge	−0.178^***^	0.099^***^	0.061^***^	0.092^***^	−0.035^*^	0.464^***^	1.000						
Lev	−0.069^***^	−0.005	−0.093^***^	0.037^**^	−0.137^***^	0.237^***^	0.369^***^	1.000					
INST	0.055^***^	0.081^***^	0.102^***^	0.086^***^	−0.051^***^	0.288^***^	0.093^***^	0.014	1.000				
Top1	−0.025	−0.005	−0.033^*^	0.056^***^	−0.034^*^	0.090^***^	−0.175^***^	−0.079^***^	0.422^***^	1.000			
ATO	−0.061^***^	−0.018	0.077^***^	0.164^***^	−0.092^***^	0.118^***^	0.156^***^	0.219^***^	0.210^***^	0.133^***^	1.000		
FIXED	−0.026	−0.051^***^	−0.054^***^	−0.027	−0.172^***^	−0.107^***^	0.131^***^	0.247^***^	−0.092^***^	−0.013	0.020	1.000	
Indus	0.188^***^	0.078^***^	−0.025	0.074^***^	−0.084^***^	0.081^***^	−0.020	−0.012	−0.054^***^	−0.154^***^	−0.002	0.064^***^	1.000

^*^P < 0.1, ^**^P < 0.05, ^***^P < 0.01.

### 4.2 Main regression results

Table 5 presents the benchmark regression results examining whether a company's drugs were negotiated successfully and its innovation investment. Column (1) shows the regression results for the independent variable nego_dum and the dependent variable RD alone, with a regression coefficient of 0.0393, which is significant at the 1% level. Column (2) adds control variables to the model, yielding regression coefficients of 0.0394, both positive and significant at the 1% level. Column (3) presents regression results after incorporating year, firm, province fixed effects, with the coefficient of 0.0109, remaining significantly positive at the 1% level. This indicates that inclusion of drugs in the NRDL through negotiation increased firms' innovation investment as a share of total assets by approximately 1.09%, compared to the sample mean of 3.08%, which supports Hypothesis 1 (H1).

**Table 5:** Benchmark regression results.

Variable	RD		
	(1)	(2)	(3)
nego_dum	0.0393^***^	0.0394^***^	0.0109^***^
	(0.0067)	(0.0061)	(0.0035)
Size		0.0006	−0.0063^***^
		(0.0013)	(0.0021)
ListAge		−0.0079^***^	0.0040^***^
		(0.0016)	(0.0015)
Lev		0.0024	−0.0046
		(0.0096)	(0.0034)
INST		0.0125^*^	0.0046
		(0.0071)	(0.0043)
Top1		−0.0179^*^	0.0212^***^
		(0.0097)	(0.0080)
ATO		−0.0036	0.0074^***^
		(0.0046)	(0.0025)
FIXED		0.0055	0.0050
		(0.0086)	(0.0053)
Indus		0.0045^***^	0.0059
		(0.0014)	(0.0043)
_cons	0.0280^***^	−0.0095	0.0918
	(0.0011)	(0.0282)	(0.0606)
Firm/Year/Province Fixed Effects	NO	NO	YES
R^2^	0.1088	0.1874	0.8666
N	3012	3012	3012

^*^P < 0.1, ^***^P < 0.01; Standard errors are in parentheses, and firm-clustered robust standard.

### 4.3 Robustness tests

#### 4.3.1 Parallel trends test

**Figure 1:**
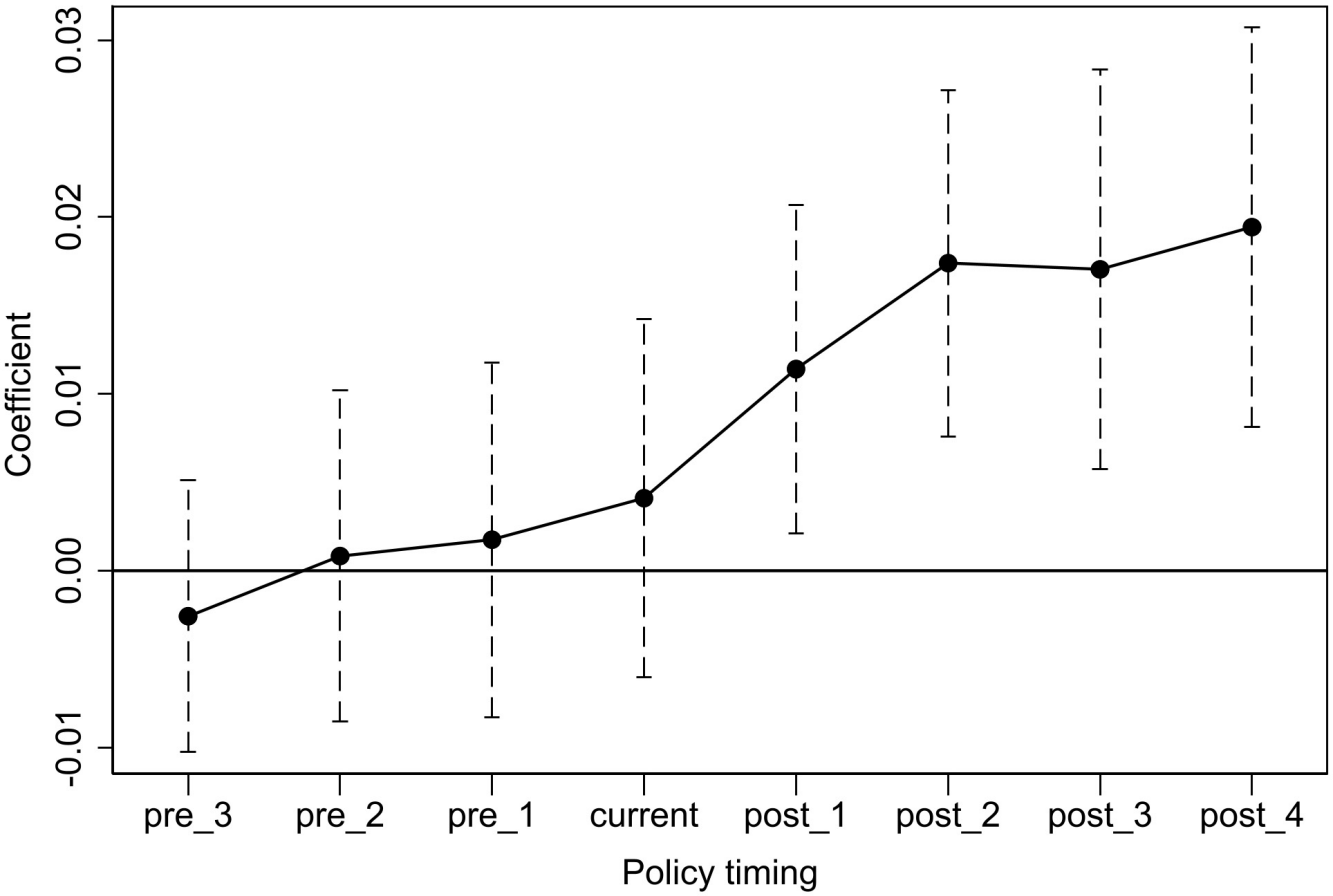
Parallel trend test.

The parallel trends test is a critical prerequisite for the validity of the DID model, requiring that the treatment and control groups exhibit identical trend lines prior to policy implementation. To address this, a series of time-period dummy variables spanning four periods before treatment to four periods after treatment (t-4 to t+4) were constructed, with t-4 serving as the reference baseline. Figure 1 displays the dynamic changes in the estimated coefficients of these dummy variables and their confidence intervals. The results indicate that, prior to the policy shock, the estimated coefficients for all periods were not significantly different from zero. This confirms that pre-treatment innovation investment did not differ significantly between the treatment and control groups, satisfying the parallel trends assumption. Meanwhile, the coefficient was not significant in the policy implementation year but became significantly positive in the first post-policy period (t+1) and exhibited an increasing trend thereafter. This not only confirms the promoting effect of negotiations on firms' innovation investment but also aligns with the entry process of drugs into hospital channels after the negotiation, indirectly reflecting “last-mile” barrier of negotiated drugs in the real-world.

#### 4.3.2 Placebo test

**Figure 2:**
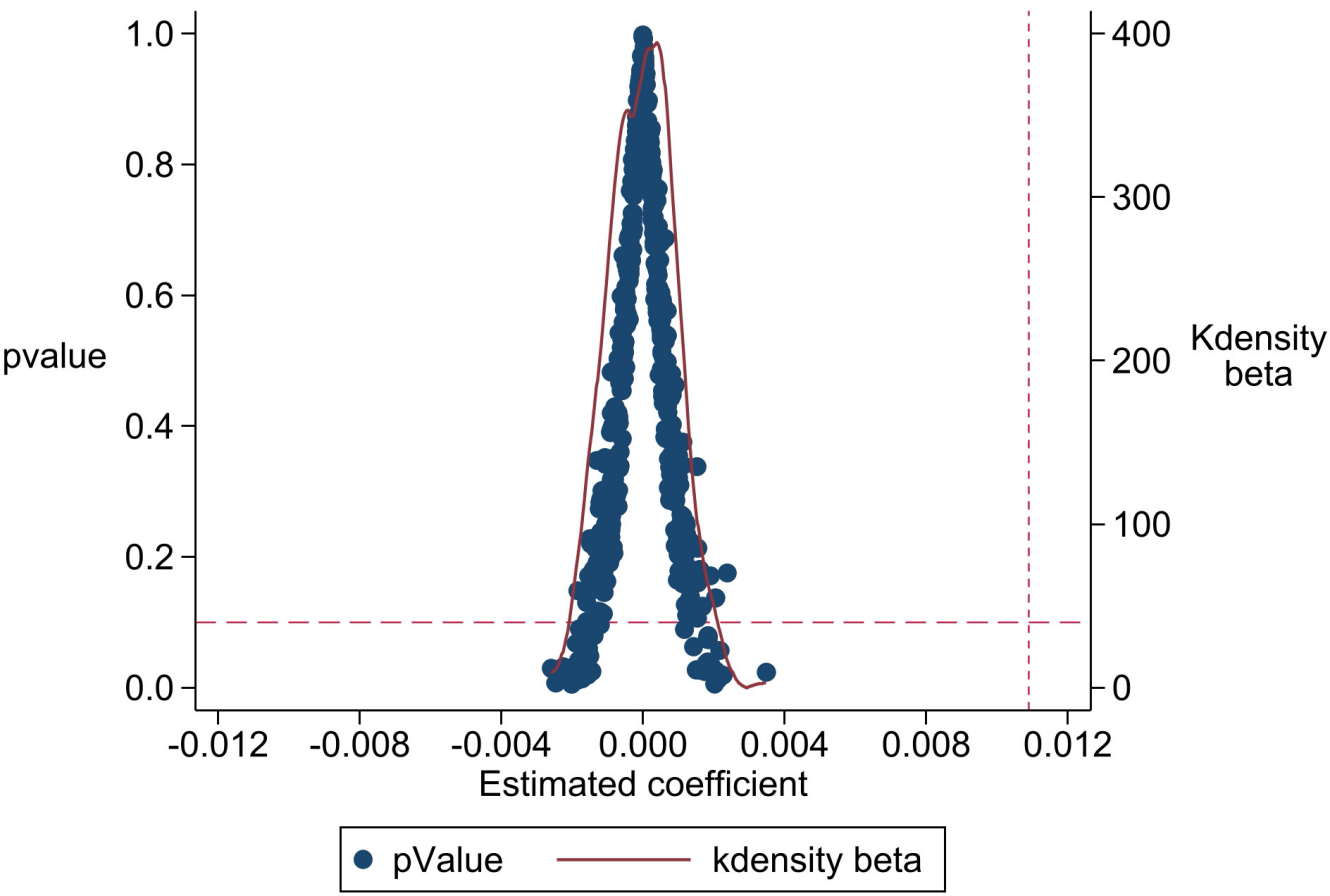
Placebo test.

To eliminate unobservable factors from interfering with the benchmark regression results, we conducted a placebo test by randomly generating a treatment group, incorporating an interaction term with the pilot implementation time into Equation (1), and repeating this process 500 times. As shown in Figure 2, the kernel density distribution of the simulated estimated coefficients is concentrated around zero, with a mean of −4.47 × 10^−5^, significantly diverging from the benchmark regression result of 0.0109, with the vast majority of coefficients failing to reach statistical significance (*P* > 0.1), thereby supporting the benchmark regression findings.

#### 4.3.3 PSM-DID

**Figure 3:**
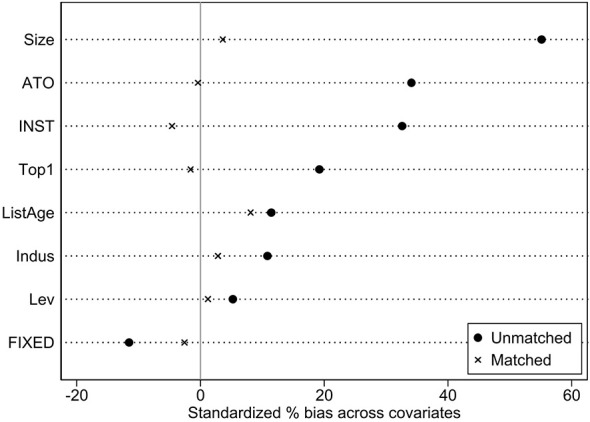
Results of balancing test.

Although the previously employed DID model mitigated endogeneity between policy implementation and corporate innovation investment to some extent, it did not fully account for potential sample selection bias. Given significant differences among firms in scale, financial status, and governance structures, inclusion of their drugs in medical insurance negotiations may not be entirely random. This could introduce systematic pre-treatment differences between the treatment and control groups prior to policy implementation, potentially undermining the validity of the DID estimates. To address this, we further incorporated propensity score matching (PSM) and constructed a PSM-DID model. Figure 3 presents the balance test results, showing a significant reduction in the standardized deviation of variables after matching, thereby indicating effective matching.

Building upon this, we further adopts the methodology applied by Brucal et al. (32) and Cole et al. (33), employing a 1:3 nearest-neighbor matching approach with a caliper value of 0.05 to select control group firms most similar to the treatment group firms. Table 6 reports the distribution of covariates in the treatment and control groups before and after matching. After matching, the bias in the covariates between the two groups was significantly reduced and no significant differences were observed.

**Table 6:** Results of balancing test.

Variable	Sample	Mean		Std. Error (%)	Reduction in absolute std. Error (%)	***T*** **test**	
		**Treatment group**	**Control group**			***T*** **value**	***P*** **value**
Size	Unmatched	22.432	21.873	55.1	93.5	13.18	0.000
	Matched	22.432	22.395	3.6		0.64	0.522
ListAge	Unmatched	2.168	2.064	11.4	29.2	2.53	0.011
	Matched	2.168	2.095	8.1		1.41	0.158
Lev	Unmatched	0.312	0.303	5.2	77.2	1.15	0.251
	Matched	0.312	0.310	1.2		0.22	0.827
INST	Unmatched	0.502	0.426	32.	85.9	7.43	0.000
	Matched	0.502	0.513	−4.6		−0.83	0.408
Top1	Unmatched	0.348	0.322	19.2	91.6	4.38	0.000
	Matched	0.348	0.350	−1.6		−0.29	0.773
ATO	Unmatched	0.616	0.519	34.1	98.7	8.07	0.000
	Matched	0.616	0.617	−0.4		−0.07	0.941
FIXED	Unmatched	0.198	0.211	−11.6	77.5	−2.55	0.011
	Matched	0.198	0.201	−2.6		−0.47	0.637
Indus	Unmatched	9.540	9.425	10.8	74.0	2.56	0.010
	Matched	9.540	9.510	2.8		0.52	0.601

The regression results for the matched samples are listed in Column (1) of Table 7. The regression coefficient of nego_dum is 0.009 and significantly positive at the 5% level, validating the robustness of the benchmark regression results. Tests indicated that the PSM-DID matched sample still satisfied the parallel trends assumption (Supplementary Figure 2).

**Table 7:** Additional robustness tests.

Variable	PSM-DID	Borusyak imputation estimator	One-period lag of independent variable	System GMM estimation	Alternative dependent variable			Considering VBP policy	Accounting for policy heterogeneity
	RD	RD	RD	RD	RDexp	RDperson	RDcap	RD	RD
	(1)	(2)	(3)	(4)	(5)	(6)	(7)	(8)	(9)
nego_dum	0.0090^**^	0.0125^***^		0.0055^**^	2.2379^***^	67.1929^*^	0.0007	0.0105^**^	0.0063^*^
	(0.0038)	(0.0031)		(0.0027)	(0.4893)	(39.2842)	(0.0013)		(0.0034)
nego_dum_t − 1_			0.0110^***^						
			(0.0037)						
RD_t − 1_				0.8185^***^					
				(0.0608)					
VBP_dum								0.0029	
								(0.0022)	
Size	−0.0083^**^	−0.0061^***^	−0.0068^**^	0.0001	1.4935^***^	221.7033^***^	−0.0009	−0.0062^***^	−0.0065^***^
	(0.0040)	(0.0017)	(0.0026)	(0.0004)	(0.2402)	(32.2777)	(0.0009)	(0.0022)	(0.0021)
ListAge	0.0067^**^	0.0031^**^	0.0026	−0.0024^***^	−0.5854^***^	−32.1550	0.0010	0.0041^***^	0.0038^**^
	(0.0026)	(0.0013)	(0.0028)	(0.0007)	(0.1769)	(23.8058)	(0.0007)	(0.0015)	(0.0015)
Lev	−0.0096	−0.0024	−0.0053	−0.0039^*^	−0.3420	−122.2878^*^	0.0026	−0.0049	−0.0057
	(0.0059)	(0.0030)	(0.0035)	(0.0022)	(0.5282)	(66.5617)	(0.0020)	(0.0033)	(0.0035)
INST	0.0062	0.0063^*^	0.0042	0.0031	0.7363	111.3447	0.0059^**^	0.0046	0.0051
	(0.0069)	(0.0037)	(0.0047)	(0.0020)	(0.5919)	(72.4021)	(0.0026)	(0.0042)	(0.0044)
Top1	0.0250^*^	0.0186^**^	0.0186^**^	−0.0049^*^	0.6915	43.7263	−0.0024	0.0211^***^	0.0218^***^
	(0.0138)	(0.0074)	(0.0093)	(0.0028)	(1.1257)	(153.9060)	(0.0043)	(0.0080)	(0.0081)
ATO	0.0061^*^	0.0077^***^	0.0088^***^	0.0054^***^	0.4521	55.8611^*^	−0.0025^**^	0.0076^***^	0.0090^***^
	(0.0036)	(0.0023)	(0.0033)	(0.0014)	(0.3254)	(33.2246)	(0.0012)	(0.0025)	(0.0026)
FIXED	0.0006	0.0064	0.0050	0.0080^***^	0.4870	84.6889	−0.0038	0.0051	0.0060
	(0.0097)	(0.0048)	(0.0055)	(0.0027)	(0.6738)	(119.2314)	(0.0029)	(0.0053)	(0.0052)
Indus	0.0085	0.0052	0.0065	0.0002	−0.2835	−133.1596	−0.0061^**^	0.0062	0.0042
	(0.0068)	(0.0039)	(0.0046)	(0.0005)	(0.7601)	(95.2141)	(0.0028)	(0.0043)	(0.0042)
_cons	0.1124		0.1009	0.0050	−28.1624^***^	−3274.7104^***^	0.0806^**^	0.0864	0.1105^*^
	(0.0985)		(0.0706)	(0.0100)	(8.7324)	(1119.7473)	(0.0315)	(0.0616)	(0.0590)
Firm/Year/Province Fixed Effects	Yes	Yes	Yes	Yes	Yes	Yes	Yes	Yes	YES
R^2^	0.8612		0.8665		0.8120	0.8993	0.6615	0.8669	0.8677
N	1682	2973	2634	2631	3012	2354	2295	3012	2903
*p*-value of AR (1) test				0.000					
*p*-value of AR (2) test				0.683					
*p*-value of Hansen test				0.154					

^*^P < 0.1, ^**^P < 0.05, ^***^P < 0.01; Standard errors are in parentheses, and firm-clustered robust standard.

#### 4.3.4 Heterogeneous treatment effects test

Given the non-simultaneous timing of the policy shock across firms, heterogeneous treatment effects may introduce bias into the estimates of the multi-period DID model. To address this, we employed robustness tests following the methods applied by Borusyak et al. (34) and Sun et al. (35) to estimate a more reliable average treatment effect. The robustness test results for Borusyak's interpolated estimates are listed in Column (2) of Table 7. The regression coefficient was 0.0125 and remained significantly positive at the 1% level. This implies that inclusion of drugs in the NRDL increased firms' innovation investment as a share of total assets by approximately 1.25%, which is broadly consistent with the 1.09% effect observed in the benchmark regression. The counterfactual analysis based on imputation further confirmed that the NDPN has a significant positive effect on firms' innovation investment.

**Figure 4:**
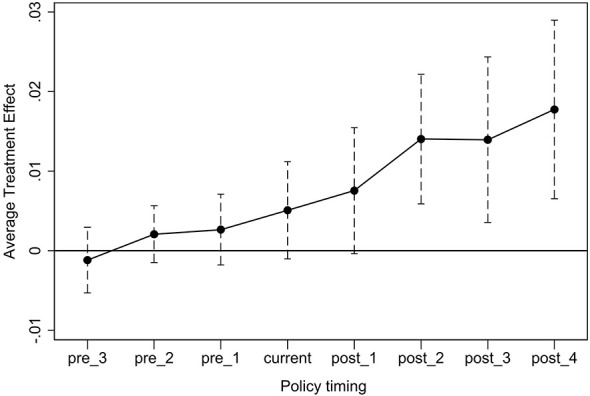
Heterogeneity-robust DID estimation.

The distribution of treated firms across negotiation years is as follows: 2016 (N = 9, 1.38%), 2017 (N = 100, 15.31%), 2019 (N = 124, 18.99%), 2020 (N = 169, 25.88%), 2021 (N = 66, 10.11%), 2022 (N = 93, 14.24%), and 2023 (N = 92, 14.09%). The estimation results from the work of Sun et al. (35) are presented in Figure 4. The dynamic effect plot shows that, prior to the policy shock, the treatment effect coefficient was not significantly different from 0. However, after policy shock, the treatment effect became significantly positive and exhibited a certain increasing trend. This result further validates that the incentive effect of the NDPN on corporate innovation investment is temporally plausible and not driven by expectations or other factors. Moreover, the effect was not significant in Post1 but became significant in Post2, reflecting the presence of a time-lag effect, while the increasing trend of the treatment effect indirectly indicates the gradual release of the policy's innovation incentive.

#### 4.3.5 Endogeneity of policy implementation

Since inclusion of drugs in the NRDL may be correlated with firms' initial innovation levels, ignoring this could bias the estimated policy effect in a standard difference-in-differences framework. To mitigate endogeneity, the explanatory variable was first lagged by one period. The regression results in Column (3) of Table 7 show that the coefficient of nego_dum_t − 1_ increased to 0.011 and remained significantly positive at the 1% level. Building on this, a generalized method of moments (GMM) estimation was conducted, with lagged dependent variables included among the controls to address potential endogeneity. As shown in Column (4) of Table 7, the coefficient of nego_dum was 0.0055 and significantly positive at the 5% level, indicating that the main findings are robust after accounting for the endogeneity of policy shock.

#### 4.3.6 Alternative dependent variables

To further verify the robustness of the baseline results, alternative dependent variable were employed. First, following Zhu et al. (1), corporate innovation investment was measured by total R&D expenditure (RDexp, in 100 million CNY). As shown in (5) of Table 7, the coefficient of nego_dum remained significantly positive at the 1% level, with a value of 2.2379, indicating that inclusion of drugs in the NRDL increased firms' average R&D expenditure by approximately 224 million CNY. Furthermore, following Cai et al. (36), the dependent variable was measured by the number of R&D personnel (RDperson). As shown in Column (6) of Table 7, the coefficient was 67.1929 and significant at the 10% level, suggesting that successful price negotiations increased the average number of R&D personnel by about 67, thereby confirming the policy's incentive effect on corporate innovation investment. Moreover, considering that R&D expenditure can be accounted for either as an expense or capitalized, firms may capitalize more R&D costs to enhance reported profit margins following substantial drug price reductions, potentially creating a spurious increase in R&D intensity. To rule out this alternative explanation, the ratio of capitalized R&D expenditure to total assets (RDcap) was used as the dependent variable. As shown in Column (7) of Table 7, the regression coefficient was not significant, indicating that the NDPN did not substantially affect firms' R&D capitalization behavior. These results mitigate concerns that R&D intensity increases were driven by accounting treatments and further confirm the robustness of the benchmark regression findings.

#### 4.3.7 Accounting for the impact of VBP

China's National Volume-Based Procurement (VBP) policy was implemented in 2018. If a firm experienced both price negotiations and VBP, the effects of the two policies could be intertwined, potentially confounding the identification of the price negotiation effect. To address this, a firm-level VBP dummy (VBP_dum) was constructed, set to 1 for the year and subsequent years if the firm had any drugs included in the national VBP list during 2018-2023, and 0 otherwise. As shown in Column (8) of Table 7, nego_dum and VBP_dum were simultaneously included in the regression, controlling for firm, year, and province fixed effects and clustering standard errors at the firm level, the coefficient of nego_dum remained significant at the 5% level, while the coefficient of VBP_dum was not significant. This result indicates that, after accounting for the VBP policy, the promoting effect of price negotiations on corporate R&D activity remained robust, further confirming the reliability of the benchmark regression findings.

#### 4.3.8 Accounting for policy heterogeneity

In 2016 and 2017, price negotiations were led by the National Health and Family Planning Commission and the Ministry of Human Resources and Social Security, respectively. After the establishment of the National Healthcare Security Administration in 2018, substantial changes occurred in negotiation rules, organizational processes, and evaluation criteria. Treating these early negotiations as equivalent to later ones could affect the robustness of the estimates due to policy heterogeneity. To address this, firms that participated in negotiations in 2016 and 2017 were excluded, and nego_dum was reassigned following the procedure described in Section 3.2. As shown in Column (9) of Table 7, the coefficient of nego_dum remained significantly positive at the 10% level, indicating that the promoting effect of price negotiations on corporate R&D activities was not driven by specific early-stage policies, further confirming the robustness of the previous results.

#### 4.3.9 Fixed-effects model for negotiated drug quantity

Replacing the explanatory variable with the continuous variable “number of drugs negotiated by the company into the NRDL (nego_count),” the fixed-effects regression results are presented in Table 8. The coefficient remained positively significant at the 1% level, edging from 0.0043 to 0.0045 after adding controls, indicating a significant positive correlation between the number of drugs successfully negotiated and its innovation investment, thereby further validating the benchmark conclusions.

**Table 8:** Fixed effects model.

Variable	RD	
	(1)	(2)
nego_count	0.0043^***^	0.0045^***^
	(0.0007)	(0.0007)
Size		−0.0061^***^
		(0.0008)
ListAge		0.0038^***^
		(0.0008)
Lev		−0.0052^**^
		(0.0025)
INST		0.0045
		(0.0030)
Top1		0.0237^***^
		(0.0053)
ATO		0.0073^***^
		(0.0016)
FIXED		0.0044
		(0.0038)
Indus		0.0043
		(0.0028)
_cons	0.0207^***^	0.0905^***^
	(0.0012)	(0.0293)
Firm/Year/Province Fixed Effects	YES	YES
R^2^	0.1607	0.2010
N	3012	3012

^**^P < 0.05, ^***^P < 0.01; Standard errors are in parentheses, and firm-clustered robust standard.

Additionally, the control group may include firms that participated in NDPN but failed the negotiation, potentially biasing the estimated effect if the observed increase in innovation investment in the treatment group partly reflects a decline in innovation investment among failed firms in the control group. Given that negotiation failure is difficult to define and measure accurately, we directly exclude 30 Class I innovative drug enterprises from the control group that were likely to have participated. After re-estimation, the coefficient remains positive and significant at the 1% level, confirming the robustness of our results (Supplementary Table 1).

### 4.4 Mechanism tests

Based on the theoretical analysis above, price negotiations may affect firms' innovation investment through two channels: first, the external signal transmission mechanism, by enhancing external capital market valuation and access to government subsidies to promote innovation investment (H2); second, the resource release mechanism, by reducing marketing intensity and reallocating the saved marketing resources to innovation investment (H3). To test these mechanisms, we adopted a three-step mediation effect test following the work of Wen et al. (37). The results are summarized in Table 9.

**Table 9:** Mechanism test.

Variable	TobinQ	Gov	SalesExp	RD		
	(1)	(2)	(3)	(4)	(5)	(6)
nego_dum	0.4631^***^	0.2002^**^	−0.0219^*^	0.0105^***^	0.0107^***^	0.0110^***^
	(0.1505)	(0.0908)	(0.0116)	(0.0034)	(0.0035)	(0.0035)
TobinQ				0.0007^**^		
				(0.0003)		
Gov					0.0010^***^	
					(0.0004)	
SalesExp						0.0041
						(0.0061)
Size	−0.9786^***^	0.6804^***^	−0.0423^***^	−0.0056^***^	−0.0070^***^	−0.0061^***^
	(0.1183)	(0.0987)	(0.0129)	(0.0021)	(0.0022)	(0.0023)
ListAge	1.0606^***^	−0.1285^*^	0.0153	0.0032^**^	0.0041^***^	0.0039^**^
	(0.1020)	(0.0677)	(0.0098)	(0.0015)	(0.0015)	(0.0016)
Lev	0.3301	0.3357	0.0421	−0.0048	−0.0049	−0.0047
	(0.3332)	(0.2386)	(0.0341)	(0.0034)	(0.0033)	(0.0033)
INST	2.9117^***^	0.2050	0.0259	0.0025	0.0044	0.0045
	(0.4122)	(0.3966)	(0.0464)	(0.0046)	(0.0043)	(0.0042)
Top1	−1.3905^**^	1.2012^**^	0.1483	0.0222^***^	0.0200^**^	0.0206^**^
	(0.6689)	(0.5195)	(0.0966)	(0.0080)	(0.0078)	(0.0083)
ATO	1.1828^***^	0.1235	−0.0333	0.0065^**^	0.0073^***^	0.0076^***^
	(0.1757)	(0.2071)	(0.0275)	(0.0027)	(0.0025)	(0.0026)
FIXED	−0.2834	0.5560	−0.0462	0.0052	0.0044	0.0052
	(0.4092)	(0.4227)	(0.0463)	(0.0052)	(0.0052)	(0.0053)
Indus	0.3668	0.1535	−0.0143	0.0056	0.0058	0.0060
	(0.3937)	(0.3980)	(0.0497)	(0.0043)	(0.0042)	(0.0042)
_cons	16.7870^***^	−0.9612	1.2397^**^	0.0792	0.0928	0.0867
	(4.5775)	(4.1066)	(0.6083)	(0.0607)	(0.0599)	(0.0632)
Firm/Year/Province Fixed Effects	YES	YES	YES	YES	YES	YES
R^2^	0.6977	0.9221	0.8123	0.8671	0.8674	0.8667
N	3012	3012	3012	3012	3012	3012

^*^P < 0.1, ^**^P < 0.05, ^***^P < 0.01; Standard errors are in parentheses, and firm-clustered robust standard.

As shown in Column (1) of Table 9, the coefficient of the explanatory variable nego_dum on the mediating variable TobinQ was 0.4631 and significant at the 1% level, indicating that inclusion of drugs in the NRDL through negotiations significantly increased firms' capital market valuation. In Column (4) of Table 9, after including TobinQ, the coefficients of nego_dum and TobinQ on corporate innovation investment (RD) were both significantly positive (0.0105 and 0.0007, respectively), suggesting that capital market valuation serves as a mediating channel through which price negotiations affect innovation investment. That is, successful negotiations convey positive signals to the market, enhance firms' market value, and thereby promote innovation investment. These results partially support Hypothesis 2 (H2).

As shown in Column (2) of Table 9, the coefficient of nego_dum on government subsidies (Gov) was 0.2002 and significant at the 5% level, indicating that price negotiations significantly increased firms' access to government funding. In Column (5) of Table 9, the coefficients of nego_dum and Gov on corporate innovation investment (RD) were both significantly positive (0.0107 and 0.0010, respectively). This suggests that government subsidies play a significant mediating role between price negotiations and corporate innovation investment: successful negotiations convey positive signals to the government, enabling firms to receive more subsidy support, which in turn promotes innovation investment. These findings fully support Hypothesis 2 (H2).

As shown in Column (3) of Table 9, nego_dum reduced marketing intensity (SalesExp) by 2.19%, significant at the 10% level. However, Column (6) indicates that SalesExp did not significantly affect innovation investment (RD), suggesting that marketing-related cost savings did not promote a greater allocation of corporate resources toward innovative activities. Several factors may explain this phenomenon: first, marketing expenses reduction alone did not create binding incentives or policies to redirect funds to innovation; second, innovation resources reallocation depends on long-term technology accumulation, talent, and capital support, so short-term cost savings are insufficient to alter firms' resources reallocation; third, uncertainty in hospital market entry (“last-mile” issue) leads firms to conserve freed cash rather than invest in high-risk, long-cycle R&D. These results imply that price negotiations alone are insufficient to naturally reallocate firms‘ resources from marketing toward R&D. Instead, coordinated policy support and capital guidance are needed to reallocate resources toward innovation investment.

### 4.5 Heterogeneity tests

#### 4.5.1 Heterogeneity effects across firm sizes

Firm size may influence the allocation of innovation resources. Therefore, firms were classified as large or small-to-medium (SMEs) based on the median total assets for subgroup regressions. As shown in Columns (1) and (2) of Table 10, NDPN significantly incentivized innovation investment for large enterprises but had no significant effect on SMEs. This reflects the fundamental differences in resource bases and risk-bearing capacities among enterprises of different scales. Large firms typically possess greater internal cash flow, more mature innovation systems, and stronger distribution networks, enabling them to absorb short-term price pressures from negotiations and leverage superior hospital access and channel advantages to quickly scale sales of negotiated drugs, thereby converting market opportunities into innovation investment. In contrast, SMEs face resource scarcity, tighter financing constraints, and limited market expansion capabilities (38). Negotiated price reductions may directly squeeze their already narrow profit margins, leading to insufficient cash flow to sustain long-term innovation investment, even after gaining market access. Such firms may reduce their innovation spending to maintain their operations.

**Table 10:** Heterogeneity analysis.

Variable	Enterprise size grouping		Market power grouping		Negotiation timing grouping	
	Large firms	Small and medium-sized firms	Firms with strong market power	Firms with weak market power	Early negotiators	Late negotiators
	(1)	(2)	(3)	(4)	(5)	(6)
nego_dum	0.0118^***^	0.0075	0.0166^***^	−0.0003	0.0096^**^	0.0145^***^
	(0.0040)	(0.0045)	(0.0051)	(0.0032)	(0.0042)	(0.0054)
Size	−0.0027	−0.0096^*^	−0.0106^***^	−0.0046^*^	−0.0068^***^	−0.0052^***^
	(0.0027)	(0.0055)	(0.0037)	(0.0025)	(0.0023)	(0.0012)
ListAge	0.0047^*^	0.0053^***^	0.0049^**^	0.0031	0.0036^**^	0.0030^**^
	(0.0028)	(0.0019)	(0.0019)	(0.0024)	(0.0016)	(0.0012)
Lev	−0.0049	−0.0021	−0.0021	0.0003	−0.0022	−0.0029
	(0.0056)	(0.0043)	(0.0061)	(0.0039)	(0.0034)	(0.0032)
INST	0.0071	−0.0077	0.0055	−0.0023	0.0053	0.0057
	(0.0057)	(0.0096)	(0.0074)	(0.0051)	(0.0045)	(0.0035)
Top1	0.0107	0.0411^**^	0.0270^*^	0.0186^**^	0.0189^**^	0.0158^**^
	(0.0108)	(0.0171)	(0.0149)	(0.0090)	(0.0079)	(0.0067)
ATO	0.0068^**^	0.0098^*^	0.0089^**^	0.0063^*^	0.0068^**^	0.0097^***^
	(0.0032)	(0.0055)	(0.0039)	(0.0037)	(0.0027)	(0.0022)
FIXED	−0.0020	0.0067	0.0081	−0.0029	0.0054	0.0045
	(0.0088)	(0.0068)	(0.0086)	(0.0062)	(0.0055)	(0.0047)
Indus	0.0082	0.0064	0.0134	−0.0001	0.0050	0.0064^*^
	(0.0059)	(0.0055)	(0.0087)	(0.0060)	(0.0042)	(0.0038)
_cons	−0.0081	0.1487	0.1148	0.1125	0.1123^*^	0.0614
	(0.0905)	(0.1304)	(0.1188)	(0.0840)	(0.0643)	(0.0454)
Firm/Year/Province Fixed Effects	YES	YES	YES	YES	YES	YES
R^2^	0.8817	0.8755	0.8648	0.8883	0.8753	0.8669
N	1491	1495	1476	1470	2725	2641

^*^P < 0.1, ^**^P < 0.05, ^***^P < 0.01; Standard errors are in parentheses, and firm-clustered robust standard.

#### 4.5.2 Heterogeneity effects among firms with different market power

The Lerner index serves as a key indicator of a firm's market power, with higher values indicating stronger pricing power. To examine how market power moderates the effect of NDPN on innovation incentives, we followed the methodology employed by Jiang et al. (39), dividing the sample into two groups based on the median Lerner index: firms with strong market power and those with weak market power. As shown in Columns (3) and (4) of Table 10, the coefficient of nego_dum was significantly positive only in the group of firms with strong market power, whereas it was insignificant in the group with weak market power. This finding aligns with Schumpeterian innovation theory. Firms with stronger market power generally have more internal resources and greater risk-bearing capacity. Stronger market influence also facilitates hospital entry, allowing them to convert market expectations into sustained innovation investment. Conversely, firms with weaker market power face resource and capability constraints, rendering the policy's incentive effects less pronounced (40). This also suggests that price negotiations may reinforce a “Matthew effect” in the pharmaceutical industry, enabling leading firms to consolidate their innovation advantage, potentially driving the sector toward monopolization or consolidation and weakening market competition over the long term.

#### 4.5.3 Heterogeneity effects of firms at different negotiation timings

Firms may strategically choose whether and when to participate in price negotiations. Based on the interval between the earliest drug approval year and the negotiation year, the average time from market launch to inclusion in the NRDL for sample firms is approximately 4 years. Using this as a threshold, we divide the sample into an “early negotiators” group (average interval < 4 years) and a “late negotiators” group (average interval ≥ 4 years). To ensure comparability across groups, both regressions employ firms that never participated in price negotiations as control group.

Columns (5) and (6) of Table 10 report the subgroup regression results. The coefficient for late negotiators is 0.0145, significantly positive at the 1% level, slightly higher than the baseline regression of 0.0109; the coefficient for early negotiators is 0.0096, significantly positive at the 5% level, but smaller than both the baseline coefficient and that of late negotiators. A supplementary robust test was conducted to further validate the difference between these coefficients (Supplementary Table 2). This may be because, compared to late negotiators, early negotiators relatively lack product market foundation and brand recognition, which may hinder hospital access and sales growth. In contrast, late negotiators have a relatively longer pre-negotiation market presence, allowing more time to prepare clinical evidence and build physician recognition, thereby enabling them to better absorb price reductions and reinvest in innovation.

This finding also aligns with the observed time-lag effect of innovation incentives detected in Figure 1 and Figure 4. We conducted additional tests and found that the incentive effect of NRDL inclusion on corporate innovation investment is consistently positive and exhibits a time-lag characteristic of gradually strengthening over time (Supplementary Table 3 and Supplementary Figure 3), implying that firms may face a short-term post-negotiation barrier to hospital access and profit recovery, which eases only gradually over time. This “last-mile” barrier leads innovative drug firms to carefully consider the timing of their negotiation participation, and it may further explain the observed difference in innovation incentive effects between the early and late negotiation groups. Specifically, early negotiators with relatively weak market foundation and brand recognition face a more uncertain post-negotiation access delay, which attenuates their innovation response relative to late negotiators.

### 4.6 Further analysis

Previous results have demonstrated that price negotiations incentivize firms' innovation investment and identified mediating channels through enhanced capital market valuation and access to government subsidies. However, in practice, some firms may engage in “innovation embellishment,” such as increasing applications for low-quality patents rather than genuine breakthrough innovations, thereby conveying a misleading signal of innovation capability to the market and government (41). This may cause R&D expenditure to overstate firms' actual technological innovation, exaggerating the policy's true incentive effect. To distinguish whether price negotiations promote substantive or strategic innovation, building on the finding that price negotiations incentivize innovation investment, we further examine firms' innovation performance along two dimensions: quantity and quality.

Following Hall et al. (42), we distinguish substantive and strategic innovation based on patent types. Invention patents aim to advance technology and establish competitive advantage, representing high-quality substantive innovation, whereas non-invention patents (including utility models and design patents) more often reflect regulatory compliance or market-oriented strategic innovation. We first examined the number of invention patent applications (PatentInv) and non-invention patent applications (PatentUd), using applications in t+1 and t+2 periods to account for the lag between innovation investment and patent applications. To assess innovation quality, patent citations were used as a proxy for technological impact, comparing invention and non-invention patents from the perspective of market recognition. Following Bradley et al. (43), we use forward citation counts of listed companies' patents. Three-year cumulative citation windows (current year plus the following 2 years) were used to measure invention patent quality (PatentInv_Cite_3t_) and non-invention patent quality (PatentUd_Cite_3t_). Following Wenjing et al. (41), all patent quantity and quality variables were transformed using the natural logarithm of one plus the original value to mitigate extreme values, and all continuous variables were winsorized at the 1% level. Regression results are presented in Table 11.

**Table 11:** Further analysis.

Variable	PatentInv_t+1_	PatentUd_t+1_	PatentInv_t+2_	PatentUd_t+2_	PatentInv_Cite_3t_	PatentUd_Cite_3t_
	**(1)**	**(2)**	**(3)**	**(4)**	**(5)**	**(6)**
nego_dum	0.0881	0.2919^**^	0.1789	0.2540^**^	0.2757^*^	0.0392
	(0.1353)	(0.1146)	(0.1398)	(0.1240)	(0.1466)	(0.1258)
Size	0.2962^***^	0.3677^***^	0.1018	0.2190^**^	0.2685^***^	0.2915^**^
	(0.0821)	(0.0927)	(0.0921)	(0.0927)	(0.0816)	(0.1210)
ListAge	0.1083	0.0132	0.1068	−0.0177	0.5877^***^	−0.0317
	(0.0763)	(0.0894)	(0.0825)	(0.0961)	(0.0945)	(0.1154)
Lev	−0.6127^**^	−0.6583^**^	−0.7513^***^	−0.6883^**^	0.2142	−0.2141
	(0.2484)	(0.2895)	(0.2561)	(0.3025)	(0.1895)	(0.3073)
INST	0.0046	−0.3321	0.0913	−0.0656	−0.1343	−0.4459
	(0.3336)	(0.3617)	(0.3299)	(0.3680)	(0.2577)	(0.3602)
Top1	0.9994^*^	0.4048	0.5945	0.5786	−0.2235	−0.1147
	(0.5735)	(0.6636)	(0.7295)	(0.6443)	(0.5475)	(0.6530)
ATO	0.0523	0.0993	0.1381	0.0465	0.1637	−0.0996
	(0.1634)	(0.1574)	(0.1727)	(0.1734)	(0.1085)	(0.1832)
FIXED	−0.4739	0.8173^**^	−0.7081^*^	0.5660	0.1474	0.3246
	(0.3425)	(0.3913)	(0.3761)	(0.4083)	(0.3517)	(0.5596)
Indus	0.2477	−0.1342	0.1474	0.0324	0.4005	−0.0425
	(0.3305)	(0.3242)	(0.3578)	(0.3501)	(0.2897)	(0.4804)
_cons	−7.1460^*^	−5.2907	−1.7353	−3.5274	−6.8939^**^	−3.1998
	(3.7345)	(3.6394)	(4.1681)	(3.8410)	(3.0323)	(5.2396)
Firm/Year/Province Fixed Effects	YES	YES	YES	YES	YES	YES
R^2^	0.6703	0.6473	0.6820	0.6566	0.9181	0.8458
N	2667	2667	2299	2299	1938	1523

^*^P < 0.1, ^**^P < 0.05, ^***^P < 0.01; Standard errors are in parentheses, and firm-clustered robust standard.

As shown in Column (2) and (4) of Table 11, price negotiations (nego_dum) had a significant positive effect on non-invention patent applications (PatentUd_t+1_), with a coefficient of 0.2919 at the 5% significance level. Extending to t+2 (PatentUd_t+2_), the coefficient remained significantly positive at the 5% level. In contrast, Columns (1) and (3) show that the coefficients for invention patent applications (PatentInv_t+1_ and PatentIn_t+2_) were not significant. These results indicate that, in terms of innovation quantity, price negotiations may induce short-term strategic innovation.

However, the conclusions differ when considering innovation quality. Column (5) of Table 11 shows that price negotiations significantly increased the 3-year cumulative citation count of invention patents (PatentInv_Cite_3t_), with a coefficient of 0.2757 at the 10% level, whereas Column (6) of Table 11 indicates no significant change in the 3-year cumulative citation count of non-invention patents (PatentUd_Cite_3t_). This suggests that, although price negotiations did not immediately increase the number of invention patents, they significantly enhanced the technological impact and market recognition of existing invention patents, improving the quality of substantive innovation.

In summary, the impact of the price negotiation policy on corporate innovation performance exhibits a divergence between strategic and substantive innovation. In terms of quantity, the policy prompted firms to engage in strategic innovation by increasing non-invention patents. In terms of quality, it effectively enhanced the technological impact of high-quality invention patents, improving substantive innovation. This finding suggests that, under policy guidance, firms may pursue some strategic innovations to cope with short-term pressures, while concentrating core resources on fewer, more competitive substantive breakthroughs, resulting in a more prudent and focused allocation of innovation resources.

## 5 Discussion

Pharmaceutical innovation serves as the cornerstone for safeguarding public health, driving industrial upgrades, and ensuring national strategic security. It directly determines whether enterprises can develop new drugs and therapies to conquer major diseases, extend patient lives, and enhance quality of life, thus fulfilling the strategic requirements of “Healthy China.” Moreover, it constitutes the fundamental driving force for enterprises to build core technological barriers, gain sustainable competitive advantage, and propel the entire biopharmaceutical industry toward higher value-added segments of the supply chain. National Drug Price Negotiation represents a critical policy under China's current healthcare system reform, serving as a key institutional arrangement to balance medical cost control with industrial innovation incentives. Assessing its impact on corporate innovation activities is central to evaluating policy effectiveness.

Using panel data of Chinese A-share listed pharmaceutical companies from 2010 to 2024, we employed a multi-period difference-in-differences model (DID) to empirically examine the effect of the National Drug Price Negotiation policy (NDPN) on corporate innovation investment. The results indicated that inclusion in the reimbursement list through National Drug Price Negotiations significantly increases firms' innovation investment, and this conclusion remains robust after a series of checks, including parallel trends, placebo tests, PSM-DID and other robustness checks, which is consistent with previous studies (1, 23). Mechanism analysis shows that price negotiations stimulate innovation primarily through the signal transmission effect. As an authoritative signal of government quality certification, the policy significantly enhances firms' capital market valuation and government subsidies, providing incremental funding while leveraging external supervision to ensure resources are allocated to innovation investment. However, the resource release effect was not effectively realized: although negotiations reduced marketing intensity, the freed financial resources did not flow into innovation investment, likely due to the lack of institutionalized incentives to direct cost savings toward innovation, combined with the long-term nature of innovation and the uncertainty of realizing sales in the “last-mile” problem, which prompts firms to reserve cash to manage risk. These findings suggest that merely reducing the marketing burden through price negotiations is insufficient to naturally reallocate firms' resources from marketing toward R&D, coordinated supports from industrial capital guidance and government subsidies are still needed to channel resources toward innovation investment, thereby expanding upon existing mechanisms such as demand-pull and talent attraction (8).

Heterogeneity analysis reveals the boundary conditions of the policy effect: the innovation-incentive effect of price negotiations is more pronounced in large firms and those with stronger market power, likely because these firms have greater internal cash flow, more extensive distribution networks, and superior hospital access, which help absorb price reductions and overcome the “last-mile” challenge (38). However, this also suggests that price negotiations may reinforce a “Matthew effect” in the pharmaceutical industry, potentially driving long-term consolidation or monopolization and weakening market competition. Moreover, regardless of whether firms choose to participate in negotiations earlier or later after drug launch, both strategies significantly stimulate innovation investment. However, the innovation incentive effect is relatively stronger for the late negotiators, possibly because early negotiators relatively lack product market foundation and brand recognition. This finding echoes the time-lag effect we have identified, suggesting that negotiated drugs may still face “last-mile” barriers to market access and sales volume expansion.

Further analyses indicate that the impact of price negotiations on firms' innovation output exhibits a pronounced structural pattern. In terms of quantity, the policy significantly increased non-invention patent applications but had no significant effect on invention patents. In terms of quality, it significantly enhanced citations of invention patents within 3 years, while the quality of non-invention patents showed no significant changes. This divergence between strategic and substantive innovation suggests that, under policy pressure, firms may pursue some strategic innovation but tend to concentrate core resources on a few more competitive technological breakthroughs, improving the quality of substantive innovation. These findings align with China's current transition from a generic-dominated industry to an innovation-driven one, providing empirical evidence that the National Drug Price Negotiation can guide innovation resources toward high-quality outputs and accelerate the shift from a “major generics producer” to a “leading innovator” in pharmaceuticals.

Based on the above findings, we propose the following policy implications. First, the National Drug Price Negotiation policy can play a positive role in promoting innovation and should be continued and improved. The negotiation mechanism, centered on “volume for price” and guided by value, should be institutionalized and normalized, with dynamic adjustments (14). Second, policy design should ensure the smooth functioning of the dual incentive channels. For the signal transmission channel, the negotiation process should remain fair, transparent, and predictable to maintain its credibility and effectiveness in conveying signals to capital markets and the government, while government subsidy mechanisms should be enhanced to stabilize firms' financing expectations. For the resource release channel, internal governance mechanisms should be embedded through contract design, and targeted funding should guide resources toward R&D, strengthening firms' internal innovation conversion capacity. Third, policy optimization should be precise and differentiated, with targeted support for small- and medium-sized innovative firms, firms with weaker market power, and newly listed innovative drugs. At the fiscal level, tiered R&D subsidies or low-interest loans can reduce cash flow pressure from price reductions. At the regulatory level, differentiated negotiation rules can provide newly launched products or those with limited market foundations with moderate price reduction relief or longer price protection periods, avoiding the “low volume, lost price” dilemma. Policymakers should also monitor potential “Matthew effects” to prevent market monopolization from undermining competition. Fourth, efforts should be made to alleviate the “last-mile” barriers for negotiated drugs, mitigating delays and weakening of policy effects. This can be achieved by improving clinical allocation policies, optimizing “dual-channel” policies, and supporting diversified payment mechanisms (e.g., commercial health insurance), thereby enhancing firms' expected innovation returns and incentives. Finally, policy design should address differentiated innovation responses. On the one hand, the trend of improving invention patent quality should be reinforced through innovation evaluation and support systems focused on technological impact. On the other hand, non-invention patents should be guided from strategic layout toward high-quality innovation, avoiding inefficient resource allocation and promoting balanced improvements in both the quantity and quality of pharmaceutical innovation. Coordinated implementation of these measures will support China's steady transition from a major generics producer to a leading innovator, promote high-quality development in the pharmaceutical industry, and contribute to the realization of the “Healthy China” strategic goals.

This study has several limitations. First, regarding sample selection, only A-share listed pharmaceutical firms were included, which may introduce survival and size biases. Whether the findings can be generalized to unlisted biotechnology startups requires further investigation. Second, in terms of model identification, although a multi-period difference-in-differences approach was employed, time-varying unobserved confounders, such as changes in internal management teams, cannot be fully ruled out. Third, regarding the measurement of innovation outcomes, this study focused on innovation investment, patent applications, and patent citations to capture strategic and substantive innovation, without differentiating whether the policy incentivized original innovation or lower-risk “me-too” and incremental drug development. Fourth, due to data limitations, actual hospital entry rates, prescription volumes, and sales of negotiated drugs could not be directly examined, leaving the “last-mile” problem open for further investigation. These limitations also point to directions for future research.

## Data Availability

The original contributions presented in the study are included in the article/Supplementary material, further inquiries can be directed to the corresponding author.
