# Supplementary material for: Impact of China's national drug price negotiation on pharmaceutical firms' innovation activities
Source: Front Public Health. 2026 May 11;14:1812550. doi: 10.3389/fpubh.2026.1812550 (PMC13199223; doi:10.3389/fpubh.2026.1812550)
Supplement: Supplementary file 1 [file Supplementary_file_1.docx]

**Supplementary files**

.
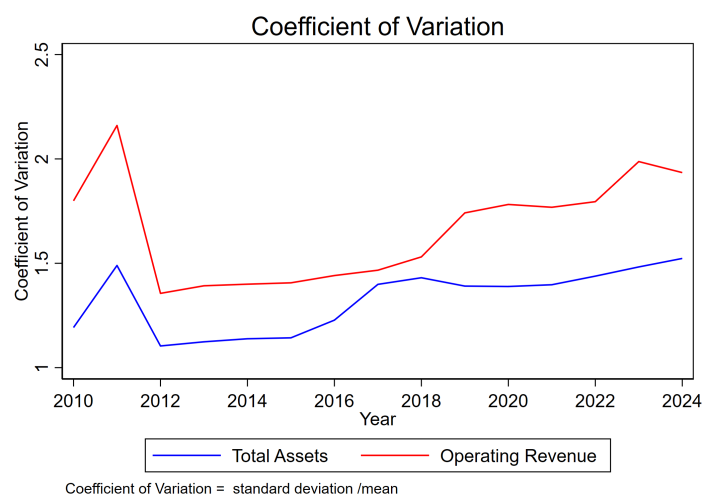


**Supplementary Figure 1: Coefficient of variation of operating revenue and total assets of sample enterprises in the observed year(s)**

**Caption to Supplementary Figure 1:** As shown in Supplementary Figure 1, the mean coefficient of variation of operating revenue for the sample firms over the observed period (1.66) is higher than that of total assets (1.32). This indicates that operating revenue exhibits greater volatility than total assets in the observed sample period.


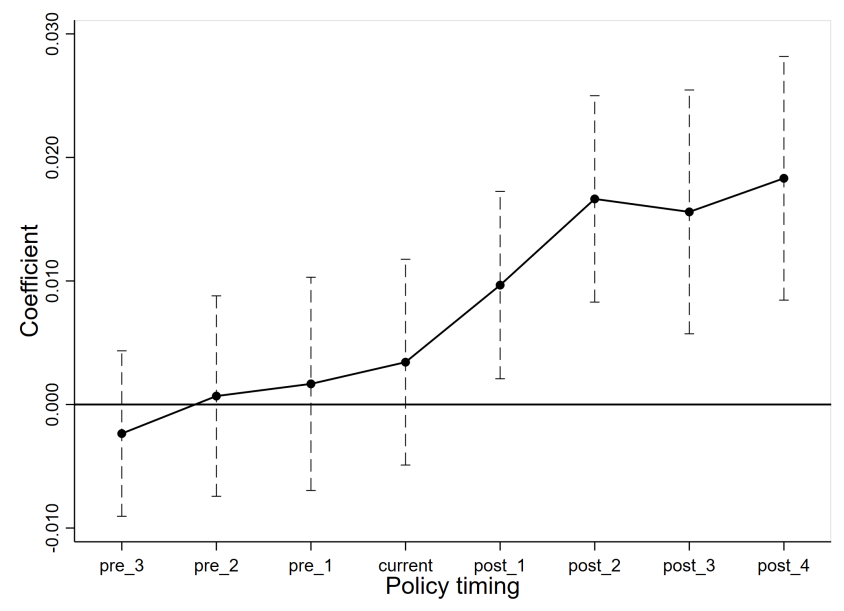


**Supplementary Figure 2: Parallel Trends Tests for PSM-DID**

**Caption to Supplementary Figure 2:** In section 4.3.3, we adopted the methodology applied by Brucal et al. and Cole et al. , employing a 1:3 nearest-neighbor matching approach with a caliper value of 0.05 to select control group firms most similar to the treatment group firms. Tests indicated that the PSM-DID matched sample still satisfied the parallel trends assumption.

**Supplementary Table 1: Robustness Test Accounting for Control Group Contamination**

| **Variable** | **Exclude the innovative drug enterprises in control group** |
| --- | --- |
|  | **RD** |
| nego_dum | 0.0112*** |
|  | (0.0035) |
| Size | -0.0059*** |
|  | (0.0023) |
| ListAge | 0.0038** |
|  | (0.0016) |
| Lev | -0.0056 |
|  | (0.0035) |
| INST | 0.0041 |
|  | (0.0046) |
| Top1 | 0.0195** |
|  | (0.0084) |
| ATO | 0.0079*** |
|  | (0.0026) |
| FIXED | 0.0037 |
|  | (0.0054) |
| Indus | 0.0053 |
|  | (0.0046) |
| _cons | 0.0880 |
|  | (0.0663) |
| Firm/Year/Province Fixed Effects | Yes |
| R^2^ | 0.8461 |
| N | 2777 |

Notes: *P<0.1, **P<0.05, ***P<0.01; Standard errors are in parentheses, and firm-clustered robust standard

**Caption to Supplementary Table 1:** Whether the control group includes firms that failed in the negotiation may lead to capturing the negative shocks suffered by the control group. Since the official authority did not disclose which firms failed in the negotiation, we manually identified 30 Class I innovative drug enterprises in the control group that were likely to have participated in the negotiation and exclude them, retaining only those that were never eligible for negotiation nor ever participated in it, including generic drug enterprises, traditional Chinese medicine enterprises, and pharmaceutical supporting enterprises. As shown in Supplementary Table 1, the coefficient increased from 1.09% to 1.12% and remains significant at the 1% level. Compared with the coefficient of 0.0109 in the baseline regression, this represented an increase, further validating the robustness of the baseline results.

**Supplementary Table 2: Regression Results of the Effect of Negotiation Time Groupings on Innovation Investment**

| **Variable** | **RD** |
| --- | --- |
| Early_negotiators | 0.0092** |
|  | (0.0044) |
| Late_negotiators | 0.0122** |
|  | (0.0057) |
| Size | -0.0063*** |
|  | (0.0021) |
| ListAge | 0.0040*** |
|  | (0.0015) |
| Lev | -0.0048 |
|  | (0.0034) |
| INST | 0.0050 |
|  | (0.0044) |
| Top1 | 0.0215*** |
|  | (0.0082) |
| ATO | 0.0073*** |
|  | (0.0026) |
| FIXED | 0.0051 |
|  | (0.0053) |
| Indus | 0.0058 |
|  | (0.0041) |
| _cons | 0.0914 |
|  | (0.0601) |
| Firm/Year/Province Fixed Effects | Yes |
| R^2^ | 0.8664 |
| N | 3012 |

Notes: *P<0.1, **P<0.05, ***P<0.01; Standard errors are in parentheses, and firm-clustered robust standard

**Caption to Supplementary Table 2 :** To further identify how the innovation incentive effect of NRDL inclusion varies dynamically with firms' negotiation timing, we divide the treatment firms into early negotiators (time lag < 4 years) and late negotiators (time lag ≥ 4 years) based on the sample average of 4 years, and construct the following model (2):

$\text{RD}_{\text{it}}\text{=}\text{α}_{\text{0}}\text{+}\beta_{1}\mathrm{Early}_{\mathrm{it}}\text{+}\beta_{2}\mathrm{Late}_{\mathrm{it}}\text{+τ}\text{X}_{\text{it}}\text{+}\text{λ}_{\text{i}}\text{+}\text{θ}_{\text{t}}\text{+}\mu_{p}\text{+}\text{ε}_{\text{it}}\text{ }$ (2)

where subscripts i and t denote firm and year, respectively. $\text{RD}_{\text{it}}$ is the dependent variable, indicating firm i’s innovation investment in year t. $\mathrm{Early}_{\mathrm{it}}$ is a dummy variable that equals 1 if the firm’s drug was firstly included in the NRDL through negotiation and the time from market launch to inclusion is less than 4 years (early negotiators), and 0 otherwise. $\text{Late}_{\text{it}}$ equals 1 for firms with a time lag of 4 years or more (late negotiators), and 0 otherwise. The coefficients $\text{β}_{\text{1}}$and $\text{β}_{\text{2}}$capture the innovation incentive effects for early and late negotiators, respectively. $\text{X}_{\text{it}}$ constitutes a set of control variables.$\text{λ}_{\text{i}}\text{, }\text{θ}_{\text{t}}\text{,}\mu_{p}$denote firm fixed effect, year fixed effect, and province fixed effect respectively. $\text{ε}_{\text{it}}$ represents the random error term. $\text{α}_{\text{0}}$ is the intercept term. Standard errors were clustered at the firm level to account for potential serial correlation and heteroskedasticity.

As is shown in Supplementary Table 2, the coefficient for early negotiators is 0.0092, which is significantly positive at the 5% level, indicating that NRDL inclusion significantly promotes innovation investment for firms that negotiate shortly after drug launch. The coefficient for late negotiators is 0.0122, also significantly positive at the 5% level, and notably larger than that of early negotiators. This suggests that the innovation incentive effect is stronger for firms with a longer pre-negotiation market presence (≥4 years). The magnitudes imply that late negotiators increase their R&D-to-assets ratio by approximately 1.22%, compared to 0.92% for early negotiators.

**Supplementary Table 3: Regression Results of Time-lag Effect of National Drug Price Negotiations on innovation investment**

| **Variable** | **RD** |
| --- | --- |
| Early_effect | 0.0066** |
|  | (0.0031) |
| Middle_effect | 0.0127*** |
|  | (0.0043) |
| Late_effect | 0.0150** |
|  | (0.0075) |
| Size | -0.0064*** |
|  | (0.0021) |
| ListAge | 0.0041*** |
|  | (0.0015) |
| Lev | -0.0044 |
|  | (0.0033) |
| INST | 0.0047 |
|  | (0.0043) |
| Top1 | 0.0205** |
|  | (0.0081) |
| ATO | 0.0076*** |
|  | (0.0026) |
| FIXED | 0.0049 |
|  | (0.0053) |
| Indus | 0.0061 |
|  | (0.0042) |
| _cons | 0.0914 |
|  | (0.0603) |
| Firm/Year/Province Fixed Effects | Yes |
| R^2^ | 0.8672 |
| N | 3012 |

Notes: *P<0.1, **P<0.05, ***P<0.01; Standard errors are in parentheses, and firm-clustered robust standard


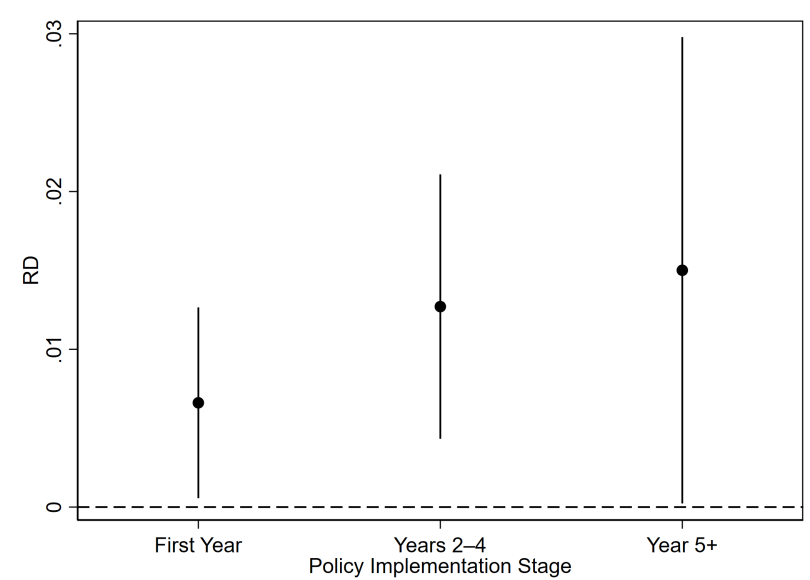


**Supplementary Figure 3: Trend of innovation investment across Different Stages after National Drug Price Negotiation**

**Caption to Supplementary Table 3 and Supplementary Figure 3:** We classified the sample by the number of years after drug negotiation, defining 1 year post-negotiation as Early_effect, 2–4 years post-negotiation as Middle_effect, and 5 years or more post-negotiation as Late_effect. We included all three variables in the same regression model (3).

$\text{RD}_{\text{it}}\text{=}\text{α}_{\text{0}}\text{+}\beta_{1}{Early\_effect}_{\mathrm{it}}\text{+}\beta_{2}{Middle\_effect}_{\mathrm{it}}\text{+}\beta_{3}{Late\_effect}_{\mathrm{it}}\text{+τ}\text{X}_{\text{it}}\text{+}\text{λ}_{\text{i}}\text{+}\text{θ}_{\text{t}}\text{+}\mu_{p}\text{+}\text{ε}_{\text{it}}\text{ }$ (3)

where subscripts i and t denote firm and year, respectively. $\text{RD}_{\text{it}}$ is the dependent variable, indicating firm i’s innovation investment in year t. ${\mathrm{Early}\_effect}_{\mathrm{it}}$ is a dummy variable equal to 1 if the firm’s drug is in the first year after successful negotiation, and 0 otherwise;${Middle\_effect}_{\mathrm{it}}$ is a dummy variable equal to 1 if the firm’s drug is in years 2–4 after successful negotiation; ${\text{Late}\text{\_effect}}_{\text{it}}$ is a dummy variable equal to 1 if the firm’s drug is in year 5 or more after successful negotiation, and 0 otherwise; $\text{X}_{\text{it}}$ constitutes a set of control variables. $\text{λ}_{\text{i}}\text{, }\text{θ}_{\text{t}}\text{,}\mu_{p}$denote firm fixed effect, year fixed effect, and province fixed effect respectively.$\text{ε}_{\text{it}}$ represents the random error term. $\text{α}_{\text{0}}$ is the intercept term. Standard errors were clustered at the firm level to account for potential serial correlation and heteroskedasticity.

As shown in Supplementary Table 3, the regression coefficient of Early_effect is 0.0066, Middle_ effect is 0.0127, and Late effect is 0.0150, indicating that the incentive effect of NDPN on corporate innovation investment was significantly positive throughout the policy implementation cycle, and presented a time-lag characteristic of gradual strengthening over time. This conclusion was further verified by the visualization results in Supplementary Figure 3, the mean coefficient across the three policy implementation stages continued to rise as the policy advances.

This confirms that the NDPN delivers far-reaching, sustainable innovation incentives for the pharmaceutical industry, supporting its long-term implementation. To further boost firms’ innovation motivation, it is necessary to address the "last-mile" dilemma of the policy, continuously strengthen supporting policies to facilitate the rapid access of negotiated drugs, so as to better enhance enterprises' innovation enthusiasm.
